# VirusLab: A Tool for Customized SARS-CoV-2 Data Analysis

**DOI:** 10.3390/biotech10040027

**Published:** 2021-11-06

**Authors:** Pietro Pinoli, Anna Bernasconi, Anna Sandionigi, Stefano Ceri

**Affiliations:** 1 Dipartimento di Elettronica, Informazione e Bioingegneria, Politecnico di Milano, 20133 Milano, Italy; pietro.pinoli@polimi.it (P.P.); stefano.ceri@polimi.it (S.C.); 2 Quantia Consulting S.r.l., Mariano Comense, 22066 Como, Italy; anna.sandionigi@quantiaconsulting.com

**Keywords:** SARS-CoV-2, data integration, virus sequencing, mutation analysis, diagnostics, COVID-19, host population

## Abstract

Since the beginning of 2020, the COVID-19 pandemic has posed unprecedented challenges to viral data analysis and connected host disease diagnostic methods. We propose VirusLab, a flexible system for analysing SARS-CoV-2 viral sequences and relating them to metadata or clinical information about the host. VirusLab capitalizes on two existing resources: ViruSurf, a database of public SARS-CoV-2 sequences supporting metadata-driven search, and VirusViz, a tool for visual analysis of search results. VirusLab is designed for taking advantage of these resources within a server-side architecture that: (i) covers pipelines based on approaches already in use (ARTIC, Galaxy) but entirely cutomizable upon user request; (ii) predigests analysis of raw sequencing data from different platforms (Oxford Nanopore and Illumina); (iii) gives access to public archives datasets; (iv) supplies user-friendly reporting – making it a tool that can also be integrated into a business environment. VirusLab can be installed and hosted within the premises of any organization where information about SARS-CoV-2 sequences can be safely integrated with information about hosts (e.g., clinical metadata). A system such as VirusLab is not currently available in the landscape of similar providers: our results show that VirusLab is a powerful tool to generate tabular/graphical and machine readable reports that can be integrated in more complex pipelines. We foresee that the proposed system can support many research-oriented and therapeutic scenarios within hospitals or the tracing of viral sequences and their mutational processes within organizations for viral surveillance.

## 1. Introduction

With the COVID-19 pandemic outbreak, huge efforts have been invested in sequencing the SARS-CoV-2 virus to trace its genome evolution and recognize the most recurring and impacting mutations. Many laboratories around the world face the problem of covering the end-to-end process of producing and analyzing viral sequences, starting from raw data produced by sequencing machines and ending with customizable reports describing viral sequences and their mutations. Reports should reflect specific needs and should include metadata about the viral host; an important aspect for effective data analysis is providing visual support for comparing arbitrarily selected viral populations. VirusLab (http://viruslab.quantiaconsulting.com/viruslab/, accessed on 3 November 2021) is a system for analyzing the characteristics of viruses collected from given host populations (viral outbreaks, hospitals, farms); it extends and customizes data analysis and visualization methods that are used in an open-source context and offers them to organizations interested in analyzing the sequences of a given population in context for various viral species, now including SARS-CoV-2, SARS-CoV, MERS, Ebola, and Dengue.

VirusLab can process the output of a different next generation sequencing platforms (Illumina, PacBio, Oxford Nanopore); in this case, the input is raw data in FASTQ format. Each interaction with VirusLab is packaged as batches of raw data material provided for a given set of input samples, hereon referred to as the *population of sequences*, which is the subject of a specific round of analysis. The raw data analysis and metadata adaptation is performed offline and requires a customization so as to take into consideration the specific features of the sequencing process and metadata design; it produces, as output, a set of sequences in FASTA format.

Otherwise, VirusLab directly uses, as input, FASTA-formatted files of SARS-CoV-2 consensus sequences. From this input, a standard pipeline produces both individual and aggregate information of the submitted viral population: the nucleotide and amino acid sequences, their phylogenetic classification (lineage [1]), their mutations, and the impact of specific mutations in clinical and virulence terms. This data analysis can take few minutes or longer time depending on the number of sequences.

Once completed, users connect to an interactive dashboard from which they can inspect data about individuals or the complete population, with indications or mutations (associated with clinical severity or virulence). Selected information is produced at the end of the session and reported to the user; once a session is closed, the system does not keep information about the submitted population; however, users may download the session state to their local machine for future re-upload and inspection.

The system is customized product is owned by Quantia Consulting, a startup specializing in data science consulting, now diversifying its business towards bioinformatics services and seeing the opportunity to enter this gigantic market by using an innovative product. Raw data analysis requires customer-specific processes that can be agreed with Quantia Consulting, possibly requiring an installation of VirusLab on the customers’ local premises. For consensus sequence analysis, instead, we allow users also to perform it directly on our endpoint.

VirusLab is targeted to health-related organizations such as hospitals, interested in adding information of the virus to clinical health records for improved care or for participation to research protocols, or biotech companies, e.g., offering zooprophylactic analyses to the farming industries or studying the virus properties for testing new drugs (VirusLab is commercialized by Quantia Consulting, a startup specializing in bioinformatics services and data science consulting).While VirusLab can be installed within secure premises so as to enable integration with sensible data (e.g., clinical information about patients), we also recommend that FASTA sequences as produced by VirusLab be submitted to public repositories, such as the one hosted by GenBank [2], so as to contribute to the global knowledge about the SARS-CoV-2 virus.

## 2. Related Work

Some companies produce systems that inspired the design of VirusLab; a detailed comparison with their functionalities is not possible, as their use is subject to limitations. Among them, BioInfoExperts (https://bioinfox.com/, accessed on 3 November 2021) offers computational methods to track the spread of viruses and bacteria; they study the longitudinal changes of viruses within the same individual, intending to identify clinically relevant patterns that predict disease onset and severity. They developed FoxSeq, a cloud-based bioinformatics software solution that enables “Precision Epidemiology”; the platform combines whole-genome pathogen sequences and integrates them with hospital electronic health record systems to improve infection control, surveillance, and patient outcomes.

The Viral Zoonoses Research Unit of Helsinki University (https://www.helsinki.fi/en/researchgroups/viral-zoonoses-research-unit/services, accessed on 3 November 2021) targets emerging and re-emerging infections and particularly viral zoonoses studying their evolution, epidemiology, diagnostics and host-virus interactions from molecular to population levels. They offer services for Virome sequencing, Genome sequencing and Meta-genome sequencing with Bioinformatics analysis of genomes/meta-genomes/virome, viral diagnostics antigens/tests, pathogen detection. Sequentia Biotech SL (https://www.sequentiabiotech.com/, accessed on 3 November 2021) offers bioinformatics software as a service for healthcare, pharma, agriculture, industry, and environment in an international environment.

A considerable number of open source/publicly available systems are dedicated to the search [3] and analysis [4,5] of viral sequences and their variants. Among them, CoV-GLUE [6] and 2019nCoVR [7] also allow to process user-input sequences and annotating them with variants and other features. Others are specialized in interactive visualization but only of pre-imported data from the GISAID database [8] (see CoVMT [9], GESS [10], outbreak.info [11], and CoV Genetics [12]). Table 1 reports the comparison of VirusLab with these systems along different features regarding the ‘input’ (whether or not user input is accepted and if viruses different from SARS-CoV-2 are handled); the ‘variation analysis’ (on the nucleotide and amino acid levels, co-occurrence of mutations on same sequences, the integration with external knowledge curated from literature); the ‘visualization’ (by defining different groups of sequences, showing mutations on a genome track, their evolution over time, also in comparative ways). To the best of our knowledge, VirusLab provides the richest support in these aspects; most overviewed systems do not allow customization of their analysis; moreover, they all process consensus sequences or are directly based on the computed variants, without providing any support from raw sequences ingestion and processing, whereas VirusLab offers offline support also for this part.

Accordingly, we report commonly used pipelines for sequence analysis, some including de novo assembly, others only variant calling and annotation. MicroGMT [13], CorGAT [14] and Coronavirus Typing Tool [15] perform sequence characterization. Galaxy recently built a rich suite of SARS-CoV-2 specific tools available at https://covid19.galaxyproject.org/ (accessed on 3 November 2021, [16]). Others community-curated efforts pre-existed the COVID-19 pandemic and they were dedicated to viruses in general, e.g., NextStrain [17], iVar [18], and viralrecon/nf-core [19]. VirusLab pipeline is more essential than these and directly employ ArticNetwork and Galaxy [20,21]; however it is readily adaptable to customers’ needs and accommodates additional modules with minimal efforts.

## 3. System Overview

**Figure 1:**
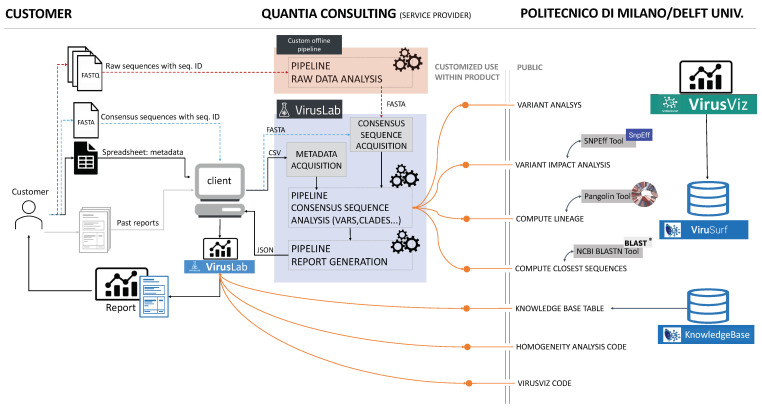
VirusLab architecture. The left part describes users interaction, the central part illustrates the internals of the system, the right part illustrates the open-source services inherited from ViruSurf and VirusViz.

The VirusLab system architecture is described in Figure 1.

### 3.1. Open-Source Component

The contribution of open source systems is illustrated in the right part of the figure. ViruSurf [22], available at http://gmql.eu/virusurf/ (accessed on 3 November 2021), is a large public database of viral sequences with integrated and curated metadata from heterogeneous sources (RefSeq [23], GenBank [2], COG-UK [24], and NMDC https://nmdc.cn/—accessed on 3 November 2021); it also exposes computed nucleotide and amino acid variants, called from original sequences. Given the current pandemic outbreak, SARS-CoV-2 data are collected from these sources; but ViruSurf contains other virus species harmful to humans, including SARS-CoV, MERS-CoV, Ebola, and Dengue. At the time of writing, the biggest dataset of SARS-CoV-2 sequences is maintained by GISAID [8], which implements a controlled access for its use. As a consequence, GISAID sequences cannot be exposed directl in VirusLab; however, researchers that have obtained personal access to GISAID EpiCoV database may download FASTA files and analyze them through VirusLab. The database is centered on sequences, described from their biological, technological, and organizational dimensions according to the Viral Conceptual Model [25]. In addition, the analytical dimension characterizes the sequence in terms of its annotations and variants. The web interface enables expressing complex search queries in a simple way; arbitrary search queries can freely combine conditions on attributes from the four dimensions, extracting the resulting sequences.

The open-source software, imported and used in the consensus sequence pipeline (next described), includes software for variant analysis (including impact), lineage assignment and computation of a given number of closest sequences to each input sequence. The code is part of the pipeline used by ViruSurf in order to load and annotate sequences from GenBank, COG-UK and NMCD. It includes some external software functions and libraries: SnpEff [26], Pangolin [27] and BLAST [28].

Some aspects of the visual environment at the client side, illustrated in Figure 1, use a customization of the open-source visualization tool VirusViz. The client also imports tables from the CoV2K knowledge base [29], which reports the most important findings about viral mutations, as soon as they are discovered and published in research articles.

### 3.2. User Interaction

User interaction is described in the left part of Figure 1. We assume that a user organization is interested in analyzing sequences of the viruses hosted by specific populations; sequences are either presented to the raw data analysis in the form of raw output from a sequencing laboratory or already presented in a so-called consensus format. We also expect users to provide metadata describing hosts, that in the case of humans may include demographic information (sex, race, ethnicity, age) as well as known co-morbidities. A user could also provide data saved from previous interactions with the tool itself (i.e., past reports); in such case, the initialization work made by the system in earlier iterations is saved and reused.

When the analysis starts from data (either raw or consensus) the user provides the input data files by some agreed data sharing tool and agrees upon data analysis parameters; we expect the first interaction to be most laborious, while subsequent interactions will use a standardized protocol.

### 3.3. VirusLab Pipelines

When data is received, it is analysed by the pipelines that constitute the main modules of the system, next illustrated in the ‘Methods’ section; the first pipeline for raw data analysis produces the consensus sequences, henceforth it is not performed when the input has already this format. The subsequent pipeline is standard and produces an output file, written in JSON, which contains all the information required at the client side for data inspection, visualization, and report generation. The user is informed when pipelines are completed and can then interact with the visual tool.

## 4. Methods

### 4.1. Raw Data Analysis

**Figure 2:**
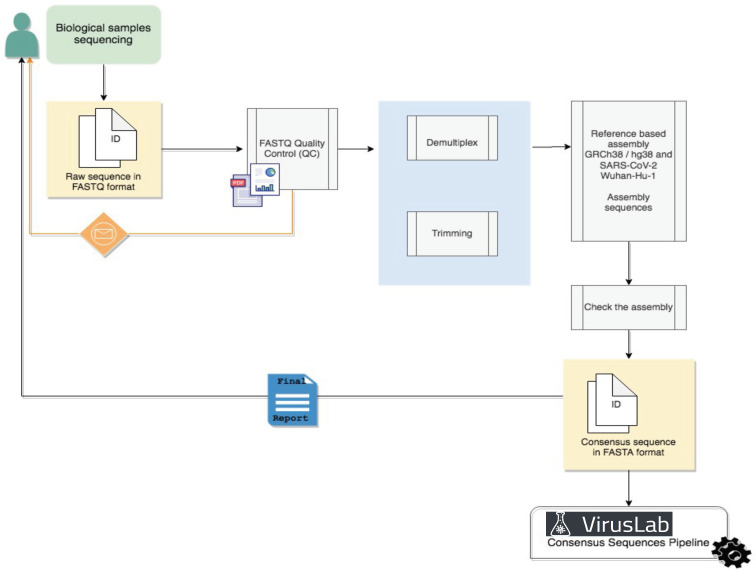
Raw sequence analysis pipeline.

The Raw Data Analysis pipeline, illustrated in Figure 2, are based on approaches already in use (ArticNetwork pipeline for Oxphord Nanopore Data [20] and Galaxy pipeline for Illumina Sequencing data [21]) but entirely cutomizable upon user request; in many cases this step is not required as the user can provide FASTA sequences directly as input. For pilot cases, we develop a prototype pipeline applicable to Illumina raw data, the most widespread technology currently for the sequencing of genomic data.

Demultiplexed reads will be generated from raw sequencing base call files and mapped to the reference SARS-CoV-2 genome (GenBank accession number: NC_ 045512.2). Alignment statistics, such as coverage and mapped reads, will be generated. For downstream analysis, a general quality control metric will be included to ensure that assembled SARS-CoV-2 genomes have at least 20x average coverage (sequencing reads >Q30) across most nucleotide positions.

### 4.2. Consensus Sequence Pipeline

**Figure 3:**
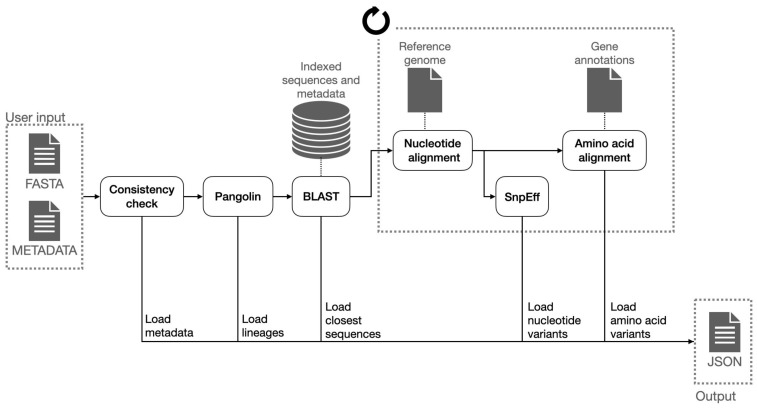
Consensus sequence analysis pipeline.

The consensus sequence analysis pipeline is responsible of producing the mutations (also called variants) with respect to the reference sequence both for what concerns RNA nucleotides and amino acids, the latter being most relevant for their predictive roles relative to key aspects such as clinical impact or altered virulence of viruses with the variants. The consensus analysis is triggered by the VirusLab web interface, after the user has uploaded the two inputs, namely the sequences and the metadata. The pipeline is schematized in Figure 3.

The Consensus sequence analysis pipeline takes as input a FASTA file with one or more consensus sequences of the same virus and a CSV file with the metadata associated to those sequences and produces as output a JSON file with all the information to be visualized by the user interface (control panel) and to be included in the report. Such information comprises:

- Lineage assignment for each sequence (for Sars-CoV-2 sequences). This operation is performed using Pangolin, a third-party open source software, which is considered the *de facto* standard tool for lineage assignment of Sars-CoV-2 sequences;
- Nucleotide variants with respect reference sequence. In order to do so, input sequences are aligned to the reference genome of the related species; the alignment is performed with a variant of the Needleman-Wunsh algorithm [30] with affine gap penality, designed to favor gaps at ends of the sequences, as this regions are usually less conserved and more subject to sequencing errors. The aligned sequence is finally scanned and nucleotide substitutions, insertions and deletions are identified. Deletions at the very end of the input sequences are removed from the output, as those are related with incomplete sequencing rather than with actual genetic differences;
- Putative impact of each nucleotide variant. Nucleotide variants are analyzed with SnpEff and annotated with their putative effect. This tool associates to each variant the indication of its position with respect to coding and structural regions of the genome and the impact on the translated protein (e.g., synonymous mutation, frameshift mutation, etc.);
- Amino acid variants with respect to the reference sequence. The list of coding sequences is retrieved from a collection of structural annotations. Each user input nucleotide sequence is converted into the corresponding amino acid sequences (one for each coding region) and each of them is aligned with the corresponding amino acid reference sequences, using a global alignment algorithm (Needleman-Wunsch); the amino acid variants are inferred from the pairwise alignments;
- List of the N (e.g., 20) most similar nucleotide sequences found in ViruSurf database and associated metadata. those sequences are retrieved using BLASTN and sorted on the identity percentage and the e-value (i.e., the number of expected hits of similar quality that could be found just by chance).

### 4.3. Report Generation Pipeline

The report generation pipeline, driven by user interactions, continuously updates the JSON file as produced by the Consensus Analysis pipeline so as to include all partial results. The state (i.e., all the information about the submitted population) is completely included within the JSON output released to the user. Users can save reports during the client sessions and reopen then within the browser, so as to enable new analyses of the data saved at various stages of the session.

## 5. Results

**Figure 4:**
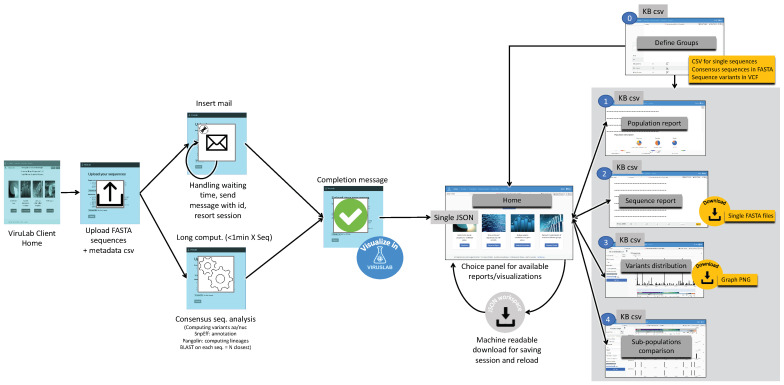
Interaction workflow for VirusLab users.

The interaction workflow with VirusLab is visualized in Figure 4. In the left part, we illustrate how a user initially interacts with VirusLab by starting the consensus sequence analysis. As the corresponding computation may take time with large populations, the user is notified when the process is completed with asynchronous communication (by email). This entire process could be fast if the customer is submitting very few sequences and the process has been fully standardized; in such case, waiting is an option.

**Figure 5:**
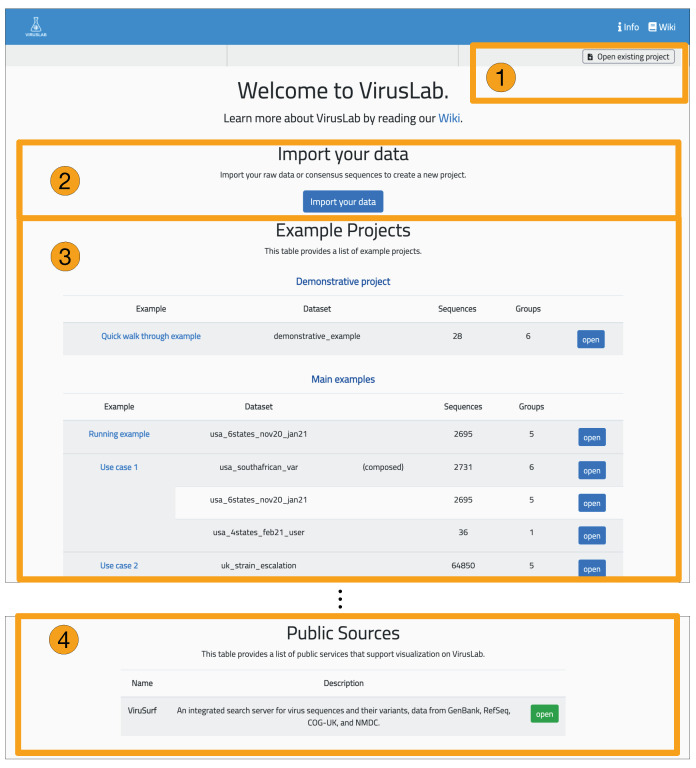
Welcome page of VirusLab.

The interaction with VirusLab starts with the welcome page (shown in Figure 5) where the user is given the possibility to (1) resort to previously saved reports in her local machine; (2) upload and process her private sequence data with corresponding metadata; (3) inspect example projects; (4) load sequences from ViruSurf.

From the landing page, the user is taken to the home page dedicated to a specific user session, acting as organizer for inspecting viral sequences produced by the consensus sequence pipeline. Interaction occurs as described in the right part of Figure 4. Starting from here, the user can:

- Manage groups: define sub-populations starting from the full set of uploaded sequences.
- Inspect the description of a population or of its groups.
- Visualize and export a sequence report, providing details on single sequences.
- Visualize the distribution of variants in the full population or its groups.
- Comparatively visualize the variants distributions of different groups, either user-defined or defined using sequences metadata.

These features are inherited from the VirusViz open tool, described in [31], however customization allows each user population to include arbitrary metadata or produce specific reports. At any time, a user may download the content in form of a multi-FASTA file, a VCF of nucleotide variants, and a CSV metadata file, useful for follow-up analysis using other platforms. As an illustrative example of outputs, we present the population report and the pages for sequence data analysis and comparative data analysis, which are also supported on VirusViz.

### 5.1. Population Report

The population report provides a summary of the characteristics of a group of sequences (either the default ‘ALL’ group, or a user-defined one), by including the following information:

- Piecharts for categorical metadata, with value and corresponding percentage on the selected group.
- Barplots for numerical or date metadata.
- Heterogeneity score indication, considering both nucleotides and amino acids.
- Table with observed nucleotide variants in the group, ordered by descending count.
- Table with observed amino acid variants in the group, ordered by descending count.
- Table with observed interesting variants in the group, present in the CoV2K knowledge base [29] with effects documented in literature.

**Figure 6:**
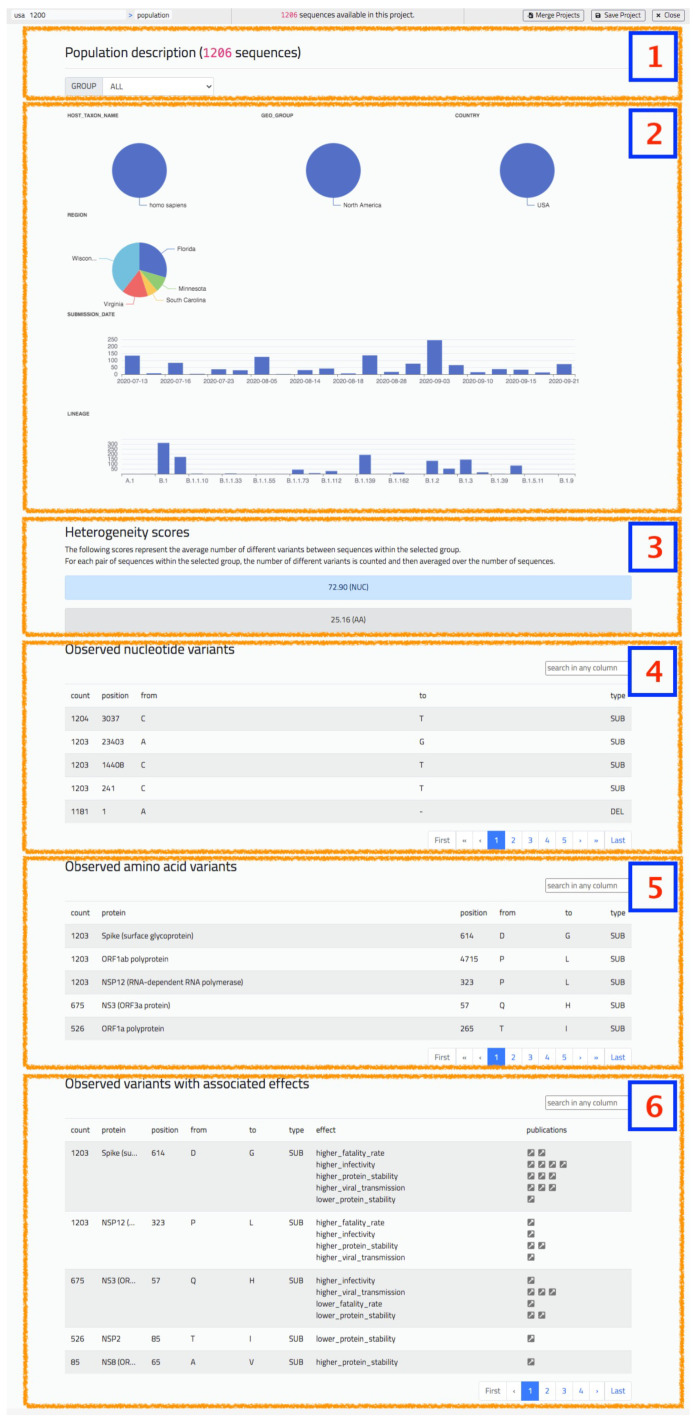
The ‘Population’ screen of VirusLab offers a general overview of characteristics of the uploaded sequences.

Tables can be searched using practical free text search box that navigates the full table in search of positions, type of mutations, or particular letters denoting nucleotides/amino acids. In the case of the last table, containing variants with associated effects, the user may also search using descriptions of the effect: for example, “higher” or “lower”, denoting the level of the effect, or “fatality_rate”, “viral transmission”, denoting the type of effect. Figure 6 shows an excerpt of a population analysis screenshot.

### 5.2. Sequence Data Analysis

**Figure 7:**
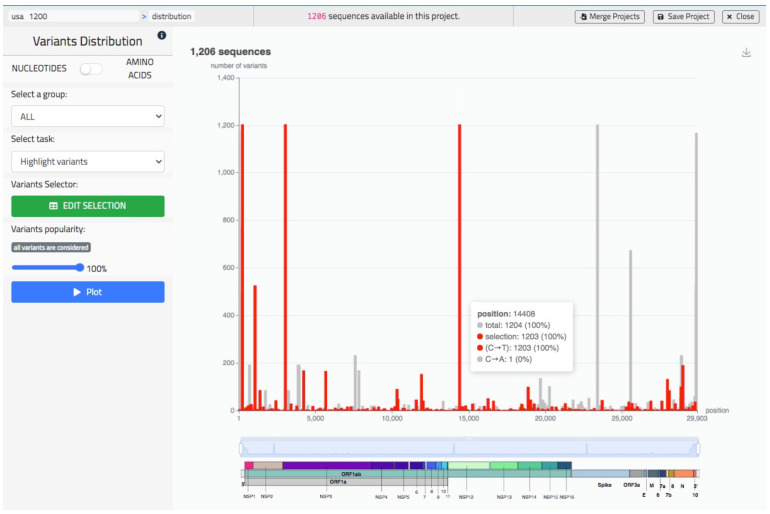
The ‘Distribution’ screen of VirusLab, a visual representation of the variants’ distribution in the uploaded sequences.

The sequence data analysis illustrated in Figure 7 provides a bar plot representing the distribution of variants, either of nucleotides or of amino acids (in the figure we represent nucleotide mutations). The specific structure of the virus sequence is shown with a colored image at the bottom of the bar plot. The user may use a slider component to zoom in a coordinate range of preference, possibly corresponding to a specific gene (colored with a unique color). By hovering on the plot, the user may visualize the specific position represented by each bar and the precise composition its corresponding count. Besides representing a default graph (bars are in black), the user may highlight a specific sequence or a specific variants (also from the knowledge base). In this case the highlighted bars are in red while other ones are in grey. See in Figure 7 where red bars represent all the C to T mutations.

### 5.3. Comparative Data Analysis

**Figure 8:**
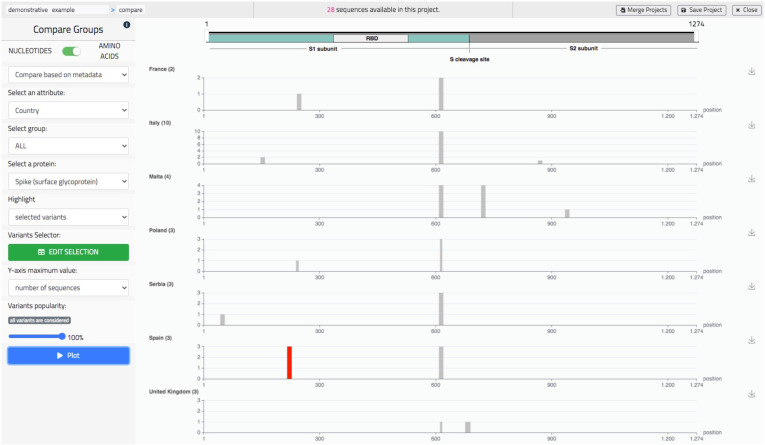
The ‘Compare’ screen of VirusLab, a comparative representation of the variants’ distributions in different groups of the observed population.

Comparative analysis allows to visually contrast the variants distributions of multiple sub-populations, either of nucleotides or of amino acids mutations, seen as tracks on the same screen. Tracks are either associated to metadata values or to groups, arbitrarily defined within a specific panel. As in the previous screen, the user is given the possibility to highlight a specific sequence or specific variants (also from the knowledge base). In Figure 8, we show an example of use of this screen representing amino acid comparing tracks based on the ‘Country’ metadata, therefore listing European countries such as United Kingdom, Italy, Malta, etc. We have selected the Spike protein and highlighted the mutation A222V, which appeared in Spain during the Summer 2020 (as studied in [32]). Several other use examples are available on the dedicated Wiki page https://github.com/quantiaconsulting/virusLab_wiki/wiki (accessed on 3 November 2021).

## 6. Discussion

The SARS-CoV-2 pandemic has changed global scenarios regarding the need to manage biological data, in particular molecular data related to the epidemiological field, not only in the academic field but also on a private level. We were inspired in our work by the emphatic reactions of visionary people that we interviewed during the Spring 2020; they reported that there was a gap in practically searching and comparing viral sequences and wished for the availability of a system such as VirusLab since the initial spreading of COVID-19. We then developed open-source tools, such as ViruSurf [22] and VirusViz [31], for searching and visually inspecting public sequences. These tools are becoming more and more relevant with the growth of the underlying data resources: public databases such as GenBank and COG-UK now store about 1.5 million of sequences, as the SARS-CoV-2 virus continuously evolves with the development of its major variants.

We soon realized, however, that there was still an uncovered gap for allowing many more organizations to support the process of analyzing viral sequences in context, e.g., with the possibility of adding sensible information to them. VirusLab covers such a gap; it has been designed so as to easily support process customization and seamless matadata integration, directly connecting data production pipelines with powerful data reporting and visualization. VirusLab can be easily installed within secure premises and used by organization interested in analyzing the SARS-CoV-2 sequences so as to enable their integration with sensible data (e.g., in hospitals, clinical information about patients; in biotech companies, information about their research processes). Although the primary purpose of VirusLab is the safe integration with protected metadata, VirusLab enables a reliable production of FASTA sequences; these can be easily submitted to public repositories such as GenBank, of course without carrying any sensible information, so as to contribute to the growth of public data about the viral evolution.

To date, the VirusLab tool is being tested for the analysis and management of SARS-CoV-2 data in monitoring projects at two companies in the biotechnology sector, i.e., FEM2 Ambiente (https://www.fem2ambiente.com/en/, accessed on 3 November 2021) and TheBioArte (https://thebioarte.com/, accessed on 3 November 2021). In the first case, the project is linked to data from environmental monitoring; in the second case, the tool is used for the tracking of virus variants distribution in the State of Malta. The system was developed as result of the collaboration between Politecnico di Milano, TU Delft, and Quantia Consulting; it was funded by the European Institute of Innovation and Technology (EIT-Digital) and can be inspected at http://viruslab.quantiaconsulting.com/viruslab/.

## Figures and Tables

**Table 1:** Comparison of open access resources for analyzing and visualizing viral sequences.

Features	CoV-GLUE	2019nCoVR	CoVMT	GESS	Outbreak	CoV Genetics	VirusLab
User-input FASTA/metadata support	✓	✓	-	-	-	-	✓
Non-SARS-CoV-2 viruses support	-	-	-	-	-	-	✓
Nucleotide analysis	✓	✓	✓	✓	-	-	✓
Amino acid analysis	✓	✓	✓	✓	✓	✓	✓
Mutation co-occurrence analysis	-	-	-	✓	✓	✓	✓
External knowledge integration	-	-	-	-	✓	-	✓
Population definition by metadata/mut/var	-	✓	-	-	✓	✓	✓
Mutations visualization on genome	-	✓	✓	✓	-	✓	✓
Mutation visualization through time	-	✓	✓	-	✓	✓	✓
Comparison of distributions	-	-	-	-	-	-	✓

## Data Availability

VirusLab is available at http://viruslab.quantiaconsulting.com/viruslab/ and documented at https://github.com/quantiaconsulting/virusLab_wiki/wiki.
